# 

**Published:** 1912-06

**Authors:** 


					XV/7,^ >
CONTENTS.
?ristol Medico-Chirurgical Society, March 13th,1912.
Introduced
ky J- Michell Clarke, M.A., M.D., F.R.C.P., and T. Lacy Firth,
M-S., F.RC.S   ...
97
Th
e Differential Diagnosis of Swelling in tha Breast.
By Charles
Mortox, F.R.C.S. ...
An p
^nemy of the People?Tuberculosis and Natural Selection.
By
S. Davies, M.D. ...
135
T"h
6 ^r^'f'cial Production of Pneumothorax in Phthisis by Injection
?t Nitrogen.
By Hubert Chitty, M.B., M.S., F.R.C.S.
I41
Not
on an Unusual Case of Hodgkin's Disease.
By E. Leonard
kEEs, M.D., and F. H. Edgeworth, M.D., D.Sc.
I5?
A p_
aSe of the Heroin Habit.
By J. Odery Symes, M.D.
153
1^ -       J J
?tes on "Some Dreams and their Significance."
By Sir George
H- Savage, M.D. F.R.C.P.
156
6v'ews of Books
i6o
E^tor:
i&l Notes
*74
Not
es on Preparations for the Sick
i8i
'he
Library of the Bristol Medico-Chirurgical Society   184
M
e?tin
gs of Societies
187
?Ca' Medical Notes
.10/
191
fitra-Qpania! Complications of Ear Disease : a Discussion at the

				

